# Serum lipid profile in systemic lupus erythematosus

**DOI:** 10.3389/fimmu.2024.1503434

**Published:** 2025-01-14

**Authors:** Jingxiu Xuan, Chaoqiong Deng, Huiqin Lu, Yan He, Jimin Zhang, Xiaoli Zeng, Yuechi Sun, Shiju Chen, Yuan Liu

**Affiliations:** ^1^ Department of Rheumatology and Clinical Immunology, the First Affiliated Hospital of Xiamen University, School of Medicine, Xiamen University, Xiamen, Fujian, China; ^2^ Xiamen Municipal Clinical Research Center for Immune Diseases, Xiamen, China; ^3^ Xiamen Key Laboratory of Rheumatology and Clinical Immunology, Xiamen, Fujian, China

**Keywords:** serum lipids, systemic lupus erythematosus, disease activity, meta-analysis, systemic review

## Abstract

**Background:**

Dyslipidemia presents in various autoimmune diseases, and the serum lipid profile in systemic lupus erythematosus (SLE) has not yet been clearly defined. This study aims to evaluate the level of serum lipids in patients with SLE.

**Methods:**

A case–control study evaluated four conventional sera lipids—total cholesterol (TC), triglyceride (TG), high-density lipoprotein (HDL), and low-density lipoprotein (LDL)—in patients with SLE compared to healthy controls (HCs). Correlations between serum lipids and clinical characteristics were analyzed in patients with SLE. A systematic review and meta-analysis were conducted to assess the epidemiology of lipid profiles in patients with SLE, and a random-effects meta-analysis was performed for data synthesis.

**Results:**

TC and TG were elevated significantly, and HDL decreased in patients with SLE compared to HCs. Elevated lipids were associated with progressive disease activity. TC, TG, and HDL were elevated in patients with SLE and were associated with decreased IgG, increased 24-h proteinuria, white blood cells (WBCs), and neutrophils. Decreased HDL and increased TG were associated with an increase in the Systemic Lupus Erythematosus Disease Activity Index (SLEDAI). Patients with SLE who took glucocorticoids (GCs) may have experienced increases in TC and TG, while those who took hydroxychloroquine (HCQ) may have experienced increases in TC and HDL. Eleven eligible studies including the present study on associations between serum lipids and SLE were reviewed by the meta-analysis. The results demonstrated elevated TC (MD = 0.85, 95% CI 0.82 to 0.89, *p* < 0.00001) and TG (MD = 0.96, 95% CI 0.94 to 0.99, *p* < 0.00001) levels in SLE, while HDL decreased (MD = −0.19, 95% CI −0.20 to −0.17, *p* < 0.00001).

**Conclusions:**

Dyslipidemia is present in SLE. There was a significant association between SLE disease activity and TC, TG, and HDL. The exact pathogenesis of metabolic disorders in SLE needs to be further addressed.

## 1 Introduction

Systemic lupus erythematosus (SLE) is an autoimmune disease involving multiple organs that occurs primarily in young women (1). The clinical manifestations of SLE are diverse and may involve multiple systems, including the circulatory, renal, and hematologic systems (2). When the kidney is involved, its clinical manifestations include nephritis or nephrotic syndrome. Notably, proteinuria is present in nephritis, and hyperlipidemia may be present in nephrotic syndrome (3). Involvements of the hematologic system include anemia, leukopenia, and thrombocytopenia (4). Cardiovascular involvement is a significant cause contributing to mortality in patients with SLE (5, 6). Moreover, it is well known that the risk factors of CVDs (cardiovascular diseases) grew with long-term corticosteroid use, such as hypertension, obesity, hyperlipidemia, and atherosclerosis (7–10). To date, the exact pathogenesis of SLE is unknown. Furthermore, it has been shown that metabolic disorders contribute to SLE due to abnormal intestinal flora, mainly including abnormalities of glucose tolerance and dyslipidemia, ultimately eliciting an increasing incidence of cardiovascular events (11, 12).

Assessment of serum lipid profile mainly includes total cholesterol (TC), triglyceride (TG), high-density lipoprotein (HDL), and low-density lipoprotein (LDL) in the clinic. Dyslipidemia embedding hyperlipidemia has also been extensively studied in the pathogenesis of CVDs (13). Dyslipidemia is also present in various autoimmune diseases. There are many investigations on the association between lipids and rheumatoid arthritis (RA). Cardiovascular events top the list of causes of death in RA (14). Patients with active RA at an early stage had obvious lipid metabolic disorders, manifested as elevated TG, LDL, and reduced HDL in the presence of comorbid inflammation, along with an increasing incidence of cardiovascular events (15, 16).

Previous studies have also investigated the association between serum lipid profile and SLE (17). The mechanisms underlying the abnormalities of lipid metabolism in patients with SLE are complicated. The possible mechanism included the formation of autoantibodies to lipoprotein lipases (LPLs), which disturbed homeostasis between pro-atherogenic lipoprotein and anti-atherogenic lipoprotein (18). The levels of TC and TG were significantly higher in patients with SLE than in healthy controls (HCs), while the level of HDL was reduced. This elevated TG and decreased HDL pattern in patients with SLE has been referred to in the literature as the “lupus pattern”, which is more pronounced in patients with higher disease activity (19, 20). Other studies have described inconsistent results for TC. A study showed that TC was decreased in patients with SLE compared to HCs (21). Studies on LDL are also controversial, with some suggesting no significant difference in LDL, while others indicated that LDL was increased in patients with SLE compared to HCs (12, 19). Thus, the serologic evidence for the association between serum lipids and SLE has not been well evaluated. The relevance of dyslipidemia for the risk of CVD development in SLE has not been consistently recognized.

To better understand the serum lipid profile of patients with SLE and the impact of serum lipids on the development of CVDs in SLE, the present study retrospectively investigated the serum lipid profile of patients with SLE. Furthermore, it explored the association between serum lipids and disease activity. A systematic review and meta-analysis, including previous studies and the present study, were also performed to assess the epidemiology of lipid profiles in patients with SLE.

## 2 Materials and methods

### 2.1 Participants

A case–control seroepidemiological study was conducted to analyze the serum lipid profile in SLE. A total of 203 patients with SLE admitted to the First Affiliated Hospital of Xiamen University from February 2018 to December 2019 were included. SLE was diagnosed based on the 2019 classification criteria of the American College of Rheumatology (ACR) and the European League Against Rheumatism (EULAR) (22). The control group of 100 individuals is a normal population from the physical examination center of the hospital who had no autoimmune diseases, infectious diseases, or CVDs and has taken no medications recently, including lipid-lowering agents. The medical examination reports of the selected controls, including laboratory items and imagological examinations, were shown to be normal, their serum lipid concentrations were within the reference intervals, and their age and gender matched SLE patients. The mean age of patients with SLE was (37.05 ± 14.38) years, and the mean age of the HC group was (35.5 ± 11.8) years. There were 179 female patients with SLE (88.2%) and 90 women among HCs (90%). There were no statistical differences in age and gender between the SLE and HC groups (*p*_age_ = 0.55 and *p*_gender_ = 0.70). This study was approved by the Medical Ethics Committee of the First Affiliated Hospital of Xiamen University (approval number KY-2019-022). Written informed consent was obtained from all participants in the study.

### 2.2 Clinical assessment

The disease activity of patients with SLE was assessed using the EULAR Systemic Lupus Erythematosus Disease Activity Index (SLEDAI) (23) score: 0–4 means inactive (*n* = 110), 5–9 means mild (*n* = 41), 10–14 means moderate (*n* = 23), and ≥15 means severe (*n* = 14). The SLEDAI scores for the 203 patients with SLE were 5.9 ± 5.3. Clinical data were collected, including patients’ age, gender, medical history, clinical manifestations, medications, complications, durations of illness, and clinical laboratory results. The medication treatments of patients with SLE are detailed in **Table 1**. Sera derived from patients with SLE were stored at −80°C for antibody to dsDNA quantitation assay. IgG anti-dsDNA was detected by enzyme-linked immunosorbent assay (ELISA) according to the introduction of the kit (AESKU, Germany). Negative values: <12 IU/mL; cutoff values: 12–18 IU/mL; positive values: >18 IU/mL. IgG anti-C1q was also detected by ELISA according to the introduction of the kit (IMTEC, Germany). Positive was judged as >20 U/mL.

**Table 1:** Drug treatments of patients with SLE.

Categories of drugs	Drugs	Patients with SLE (*n* = 203)
**GCs**	Prednisone, *n* (%)	82 (40.39%)
	Methylprednisolone, *n* (%)	68 (33.49%)
	Dexamethasone, *n* (%)	4 (1.97%)
**csDMARDs**	Hydroxychloroquine, *n* (%)	137 (67.48%)
	Mycophenolate mofetil, *n* (%)	40 (19.70%)
	Methotrexate, *n* (%)	14 (6.89%)
	Leflunomide, *n* (%)	12 (5.91%)
	Tacrolimus, *n* (%)	10 (4.92%)
	Cyclophosphamide, *n* (%)	8 (3.94%)
	Ciclosporin, *n* (%)	6 (2.95%)
	Thalidomide, *n* (%)	2 (0.98%)
	Azathioprim, *n* (%)	1 (0.49%)
**bDMARDs**	Belimumab, *n* (%)	1 (0.49%)
**NSAIDs**	Aspirin, *n* (%)	27 (12.32%)
	Celecoxib, *n* (%)	11 (5.41%)
	Etoricoxib, *n* (%)	2 (0.98%)
	Imrecoxib, *n* (%)	1 (0.49%)
**LLDs**	Atorvastatin, *n* (%)	5 (2.46%)
	Pitavastatin, *n* (%)	1 (0.49%)

GCs, glucocorticoids; csDMARDs, conventional synthetic disease-modifying anti-rheumatic drugs; bDMARDs, biological disease-modifying anti-rheumatic drugs; NSAIDs, nonsteroidal anti-inflammatory drugs; LLDs, lipid-lowering drugs.

### 2.3 Detection of serum lipids

TC, TG, HDL, and LDL of all patients with SLE and HCs have been measured routinely by commercial reagents according to the instructions of the kits in the clinical laboratories of the hospitals where the serum lipid results were obtained. The reference interval for TC was 3.1–5.2 mmol/L; that for TG was 0.4–1.82 mmol/L; that for HDL was 1.04–1.55 mmol/L; and that for LDL was 0–3.1 mmol/L.

### 2.4 Statistical analysis

The raw data were organized, and outliers were verified or excluded within an interpretable range. Continuous variables in comparing serum lipid profiles between patients with SLE and HCs were expressed as mean ± standard deviation (mean ± SD). Normality tests were conducted for continuous variables. The Mann–Whitney *U* test was used for between-group comparisons because the data did not meet normal distribution. Since the serum lipid profiles had a non-normal distribution, Spearman or Spearman rank correlation was used to analyze whether serum lipids and clinical characteristics of patients with SLE were correlated and the magnitude of the correlation. Data analysis and plotting were performed using GraphPad software (Version 8.0, GraphPad Software, California, USA). Binary logistic regression analysis was conducted to assess the independence of serum lipid profile differences between patients with SLE and HCs after adjusting for gender and age. Regression coefficients β, odds ratios (ORs), and adjusted *p*-values were displayed. Through natural logarithm transformation of non-normal distribution data, multiple linear regression analysis was performed to assess the independent relationship between serum lipid profile and gender, age, disease durations, and drugs in patients with SLE, including regression coefficients β and adjusted *p*-values. Regression analysis was performed using IBM SPSS Statistics (Version 26.0, IBM Corp., Armonk, NY, USA). All tests were two-tailed, and *p* < 0.05 was considered statistically significant.

### 2.5 Systematic review and meta-analysis

A systematic review and meta-analysis were conducted to evaluate the relationship between lipid profile and patients with SLE. PubMed, Embase, and Cochrane electronic databases were searched for studies published before 10 April 2023. Reference citations of included studies were also searched to identify additional relevant articles. The following search terms were used: (“Systemic Lupus Erythematosus” OR “Lupus Erythematosus Disseminatus” OR “Libman-Sacks Disease” OR “Disease, Libman-Sacks” OR “Libman Sacks Disease” OR “Lupus Erythematosus, Systemic”) AND (“Metabolism, Lipid” OR “Lipid Metabolism”) without language restrictions. The inclusion criteria for studies in this meta-analysis were as follows: (1) cohort studies, cross-sectional studies, or case–control studies; (2) adult patients; and (3) at least 10 patients in the SLE group. The exclusion criteria were as follows: (1) letters, reviews, abstracts, case reports, or comments; (2) not HCs; (3) overlapping data, unavailable or insufficient data; and (4) patients receiving statin or fibrate drugs. A random-effect or fixed-effect model was performed to pool the mean difference (MD) with 95% CI on the association between lipid profile and patients with SLE. Heterogeneity across studies was performed by *I* ^2^ test, and an *I* ^2^ over 50% suggested high heterogeneity (24). All analyses were performed by RevMan software (Version 5.4; Cochrane Collaboration, London, UK). *p* < 0.05 was considered statistically significant.

## 3 Results

### 3.1 Case–control study

#### 3.1.1 Serum lipid profile in patients with SLE

**Figure 1:**
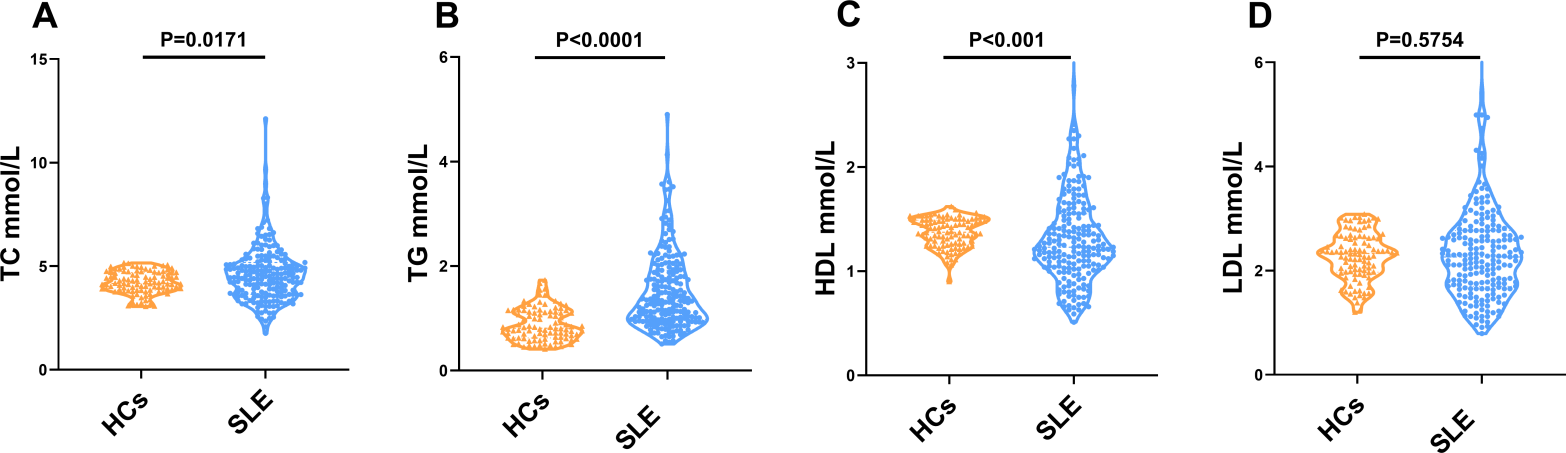
Comparison of TC, TG, HDL, and LDL concentrations between patients with SLE and HCs. **(A)** The differences in the concentration of TC between patients with SLE and HCs. **(B)** The differences in the concentration of TG between patients with SLE and HCs. **(C)** The differences in the concentration of HDL between patients with SLE and HCs. **(D)** The differences in the concentration of LDL between patients with SLE and HCs.

In the case–control study, the concentrations of 50 cases (24.63%) of TC, 55 cases (27.09%) of TG, and 31 cases (15.27%) of LDL were higher than the reference intervals. In 48 cases (23.65%), HDL was lower than the reference intervals. TC and TG concentration increased significantly in patients with SLE compared to HCs. However, HDL was markedly decreased in the SLE group compared to the HC group. Interestingly, there was no significant difference in LDL between the two groups. Therefore, LDL may not be included in later correlation analyses (**Table 2**; **Figure 1**).

**Table 2:** Serum lipid profiles of patients with SLE and HCs in the case–control study.

Serum lipids	SLE (*n* = 203)	HCs (*n* = 100)	*p*-values	Regression coefficient β	OR	Adjusted *p*-values*
**TC** (mmol/L)	4.60 ± 1.35	4.21 ± 0.53	**0.0171**	4.643	103.830	**<0.0001**
**TG** (mmol/L)	1.52 ± 0.74	0.88 ± 0.30	**<0.0001**	2.968	19.444	**<0.0001**
**HDL** (mmol/L)	1.31 ± 0.43	1.37 ± 0.14	**<0.001**	−5.569	0.004	**<0.0001**
**LDL** (mmol/L)	2.32 ± 0.88	2.27 ± 0.45	0.5754	−5.438	0.004	**<0.0001**

Bold values suggest statistically significant findings. *adjusted for age and gender.

To assess the independence of serum lipid profile differences between patients with SLE and HCs, binary logistic regression analysis was conducted, controlling for confounding factors such as age and gender. The results indicated significant differences in all serum lipids between the patients with SLE and HCs. Four indicators were the independent risks for SLE. TC (OR = 103.830, *p* < 0.0001) and TG (OR = 19.444, *p* < 0.0001) were increased in SLE. However, HDL (OR = 0.004, *p* < 0.0001) and LDL (OR = 0.004, *p* < 0.0001) decreased (**Table 2**).

**Figure 2:**
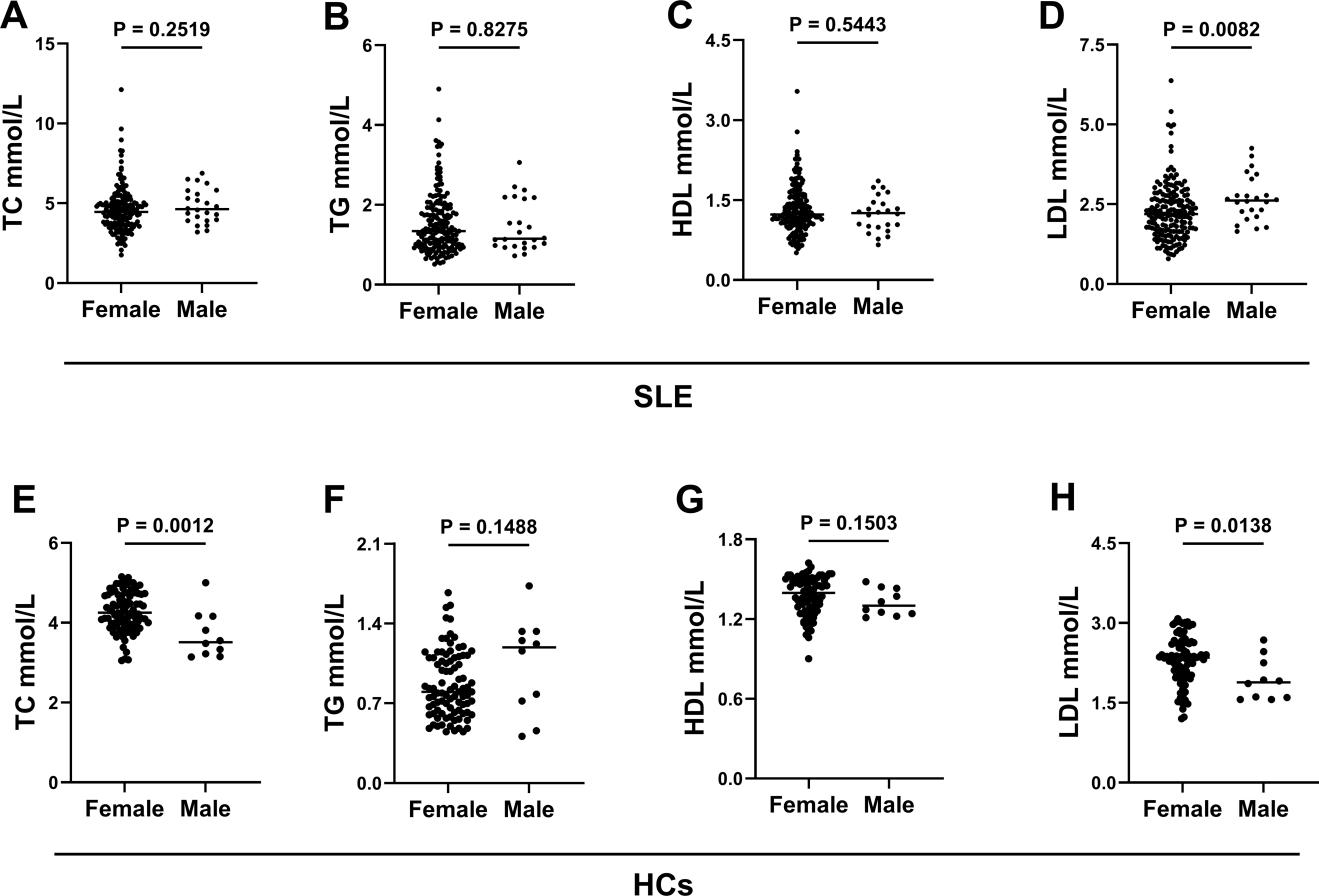
Comparison of the TC, TG, HDL, and LDL levels between women and men in patients with SLE and HCs. **(A)** The concentration differences of TC between women and men in patients with SLE. **(B)** The concentration differences of TG between women and men in patients with SLE. **(C)** The concentration differences of HDL between women and men in patients with SLE. **(D)** The concentration differences of LDL between women and men in patients with SLE. **(E)** The concentration differences of TC between women and men in HCs. **(F)** The concentration differences of TG between women and men in HCs. **(G)** The concentration differences of HDL between women and men in HCs. **(H)** The concentration differences of LDL between women and men in HCs.

Approximately 90% of female patients were seen in SLE; gender may influence the serum lipid profiles during illness. Therefore, a comparison of TC, TG, HDL, and LDL concentrations between women and men in both groups of patients with SLE and HCs was conducted. The results showed that only LDL exhibited a significant difference between male and female patients in the SLE group, with male patients having higher LDL levels than female patients. However, in the HC group, the TC and LDL levels were higher in female than in male patients, while no significant difference was observed in other lipids (**Figure 2**).

#### 3.1.2 Correlation of serum lipids with clinical characteristics in patients with SLE

**Figure 3:**
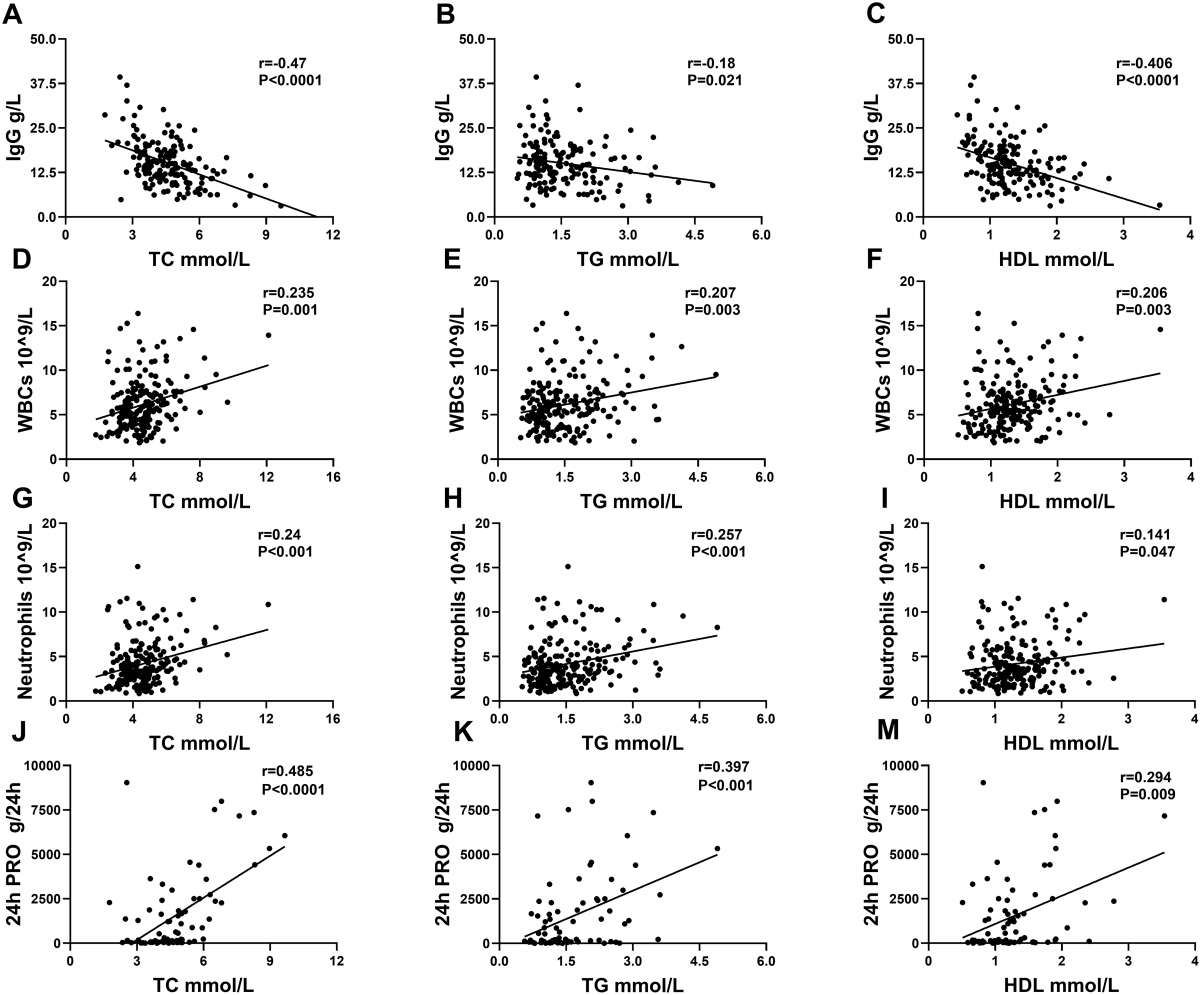
Correlation analysis between serum lipids and IgG, WBCs, neutrophils, and 24-h PRO in patients with SLE. **(A–C)** displayed a correlation between IgG and TC, TG, and HDL. **(D–F)** displayed a correlation between WBCs and TC, TG, and HDL. **(G–I)** displayed a correlation between neutrophils and TC, TG, and HDL. **(J–M)** displayed a correlation between 24-h PRO and TC, TG, and HDL.

The data showed that the serum lipid profile and clinical characteristics of patients with SLE were extremely strongly correlated. Serum IgG was especially reversely associated with the levels of TC, TG, and HDL. Elevated TC, TG, and HDL were associated with increased 24-h urinary protein quantities, white blood cells (WBCs), and neutrophils (**Table 3**; **Figure 3**).

**Table 3:** Serum lipid and clinical characteristic correlations in patients with SLE.

	TC		TG		HDL	
	*r*	*p*	*r*	*p*	*r*	*p*
**WBCs**	0.235	<0.001*	0.207	0.003*	0.206	0.003*
**Neutrophils**	0.240	<0.001*	0.257	<0.001*	0.141	0.047*
**Lymphocytes**	0.048	0.495	−0.104	0.142	0.250	<0.0001*
**PLTs**	0.225	0.001*	−0.002	0.974	0.200	0.004*
**HGB**	0.076	0.283	0.146	0.039*	0.284	<0.0001*
**IgG**	−0.470	<0.0001*	−0.180	0.021*	−0.406	<0.0001*
**IgA**	−0.210	0.013*	−0.060	0.487	−0.207	0.015*
**C3**	0.118	0.096	−0.103	0.144	0.263	<0.0001*
**C4**	0.128	0.069	−0.093	0.187	0.222	0.002*
**AST**	−0.159	0.023*	−0.063	0.371	−0.202	0.004*
**UA**	0.189	0.008*	0.230	0.001*	−0.080	0.266
**Urea**	0.184	0.010*	0.222	0.002*	0.022	0.763
**Cr**	0.096	0.180	0.255	<0.001*	0.029	0.689
**24-h PRO**	0.485	<0.0001*	0.397	<0.001*	0.294	0.009*
**PRO**	0.303	<0.0001*	0.380	<0.0001*	0.060	0.406
**ACR**	0.537	<0.0001*	0.256	0.098	0.142	0.363
**CRP**	−0.174	0.030*	0.021	0.798	−0.334	<0.0001*
**ESR**	−0.107	0.207	0.217	0.009*	−0.360	<0.0001*
**ANA**	−0.079	0.296	0.168	0.026*	−0.287	<0.001*
**Anti-dsDNA**	−0.050	0.476	0.070	0.322	−0.144	0.042*
**Anti-C1q**	−0.247	0.189	0.223	0.235	−0.574	<0.001*
**TGAb**	−0.281	0.044*	−0.102	0.471	−0.198	0.159
**SLEDAI**	−0.022	0.759	0.167	0.020*	−0.295	<0.0001*
**Disease duration**	0.095	0.183	−0.022	0.753	0.159	0.025*
**Drugs**	0.130	0.065	0.039	0.576	0.164	0.020*
**HCQ**	0.138	0.049*	−0.028	0.695	0.224	0.001*
**GCs**	0.237	0.001*	0.141	0.045*	0.107	0.129

*Bold values suggest statistically significant findings. 24-h PRO, 24-h proteinuria quantitation; PRO, proteinuria quality; ACR, Umalb/Ucr; ANA, antinuclear antibody; Anti-dsDNA, type IgG anti-double-stranded DNA antibody; Anti-C1q, anti-complement 1q antibody; TGAb, antibody to thyroglobulin; “Drugs” means untreated with medications vs. treated with medications; HCQ, hydroxychloroquine; GCs, glucocorticoids.

Not only were TC, TG, and HDL associated with the above four clinical indicators, but each of the three serum lipids was also associated with some other indicators. When the level of TC rose, UA, Urea, PRO, ACR, and PLTs increased, and TGAb, IgA, CRP, and AST decreased. A significant increase in TC was shown in patients treated with hydroxychloroquine (HCQ) and/or glucocorticoids (GCs). At the same time, TC is not different between patients with and without medications. When TG was elevated, UA, Cr, Urea, PRO, ESR, ANA, HGB and SLEDAI increased. Patients with SLE who have taken GCs also showed an increase in TG. When HDL was decreased, C3, C4, HGB, lymphocyte, disease duration, and PLTs were also decreased, whereas CRP, ESR, IgA, AST, SLEDAI, disease durations, and autoantibodies, including ANA, anti-dsDNA, and anti-C1q, were increased. Furthermore, HDL increased dramatically in both the medication and HCQ groups (**Table 3**). Overall, the disease activity got aggressive with increased TC and TG and decreased HDL. Thus, analyzing the connection between serum lipids and multiorgan damages, HDL was reversely associated with multiorgan injuries including lupus nephritis (*r* = −0.166, *p* = 0.0184), interstitial lung disease (*r* = −0.207, *p* = 0.003), gastrointestinal diseases (*r* = −0.159, *p* = 0.024), osteoarthritis (*r* = −0.198, *p* = 0.005), hematological diseases (*r* = −0.253, *p* < 0.001), and dermatological impairment (*r* = −0.217, *p* = 0.002). The remaining lipids were not significantly linked to multiorgan damage.

The correlations between dyslipidemia and clinical features were further analyzed in patients with SLE who had higher levels of TC and TG, and lower levels of HDL than reference intervals. Hypercholesterolemia was associated with elevated 24-h PRO, PRO WBCs, and neutrophils. SLEDAI scores were also increased. Disease progression got worse in patients with SLE with dyslipidemia. Hypercholesterolemia is inversely correlated with IgG, AST, ALT, and antibodies to the thyrotropin receptor (TRAbs). Hypertriglyceridemia was accompanied by elevated CRP. The negative correlation between HDL and IgG and anti-C1q antibodies became more pronounced. Similarly, TNFα increased, accompanied by decreased HDL. These results suggested that dyslipidemia development is associated with severe disease course and progressive inflammatory state in SLE (**Table 4**).

**Table 4:** Correlation analysis between serum lipids and clinical characteristics in patients with SLE with dyslipidemia.

	TC ≥ 5.2 mmol/L		TG ≥ 1.82 mmol/L		HDL ≤ 1.04 mmol/L	
	*r*	*p*	*r*	*p*	*r*	*p*
**WBCs**	**0.279**	0.049*	0.192	0.160	0.132	0.378
**Neutrophils**	**0.304**	0.032*	0.259	0.054	0.122	0.414
**HGB**	−0.119	0.412	−0.055	0.689	**0.336**	0.021*
**AST**	−**0.344**	0.014*	−0.152	0.263	0.065	0.661
**ALT**	−**0.279**	0.049*	−0.104	0.444	0.188	0.202
**IgG**	−**0.357**	0.019*	−0.183	0.209	−**0.497**	<0.001*
**24-h PRO**	**0.614**	0.003*	−0.002	0.993	0.037	0.864
**PRO**	**0.578**	<0.0001*	0.237	0.084	−0.028	0.853
**CRP**	−0.292	0.064	**0.329**	0.031*	−0.159	0.308
**TNFα**	0.373	0.323	0.357	0.432	−**0.569**	0.042*
**Anti-C1q**	0.071	0.879	−0.165	0.648	−**0.781**	0.002*
**TRAb**	−**0.867**	0.012*	−0.164	0.651	0.161	0.567
**SLEDAI**	**0.378**	0.007*	−0.097	0.489	0.112	0.465

*Bold values suggest statistically significant findings. TRAb, antibody to thyrotropin receptor.

The above data indicated a multifactorial effect on the lipid profile in patients with SLE. A multiple linear regression analysis was conducted to analyze the independence of each factor on the lipid profile. Traditional factors such as gender and age, disease duration, and medications of high-frequency use, including HCQ and GCs, which are picked out to influence the lipid profile, were conducted. The results showed that the use of GCs in patients with SLE significantly increased TC (β = 0.214, 95% CI 0.043 to 0.215, *p* = 0.004) and LDL (β = 0.253, 95% CI 0.088 to 0.313, *p* = 0.001), while the use of HCQ increased HDL (β = 0.213, 95% CI 0.046 to 0.248, *p* = 0.004). Additionally, male patients with SLE had higher LDL levels (β = 0.160, 95% CI 0.026 to 0.336, *p* = 0.022); gender difference seems to affect the lipids in SLE. However, age and disease duration did not significantly impact the lipid profile in our study (**Table 5**).

**Table 5:** Multiple linear regression analysis of the independent relationship between serum lipid profile and gender, age, disease duration, and medications in patients with SLE.

Lipids profile	Factors	Regression coefficient β	95% CI	Adjusted *p*-values
**TC**	Gender (Male)	0.077	−0.053,0.185	0.273
	Age (Y)	−0.040	−0.004,0.002	0.569
	Disease duration (Mo)	0.076	−0.0003,0.001	0.286
	Drugs			
	HCQ	0.088	−0.034,0.140	0.234
	GCs	0.214	0.043,0.215	0.004*
**TG**	Gender (Male)	−0.043	−0.252,0.135	0.553
	Age (Y)	0.017	−0.004,0.005	0.819
	Disease duration (Mo)	−0.025	−0.001,0.001	0.727
	Drugs			
	HCQ	−0.082	−0.219,0.065	0.284
	GCs	0.146	−0.001,0.280	0.052
**HDL**	Gender (Male)	−0.022	−0.160,0.116	0.756
	Age (Y)	−0.018	−0.004,0.003	0.804
	Disease duration (Mo)	0.101	−0.0002,0.001	0.155
	Drugs			
	HCQ	0.213	0.046,0.248	0.004*
	GCs	0.073	−0.049,0.151	0.318
**LDL**	Gender (Male)	0.160	0.026,0.336	0.022*
	Age (Y)	−0.012	−0.004,0.003	0.861
	Disease duration (Mo)	0.094	−0.0003,0.002	0.179
	Drugs			
	HCQ	−0.032	−0.139,0.088	0.664
	GCs	0.253	0.088,0.313	0.001*

*Bold values suggest statistically significant findings. Y, year; Mo, month.

### 3.2 Systematic review and meta-analysis

**Figure 4:**
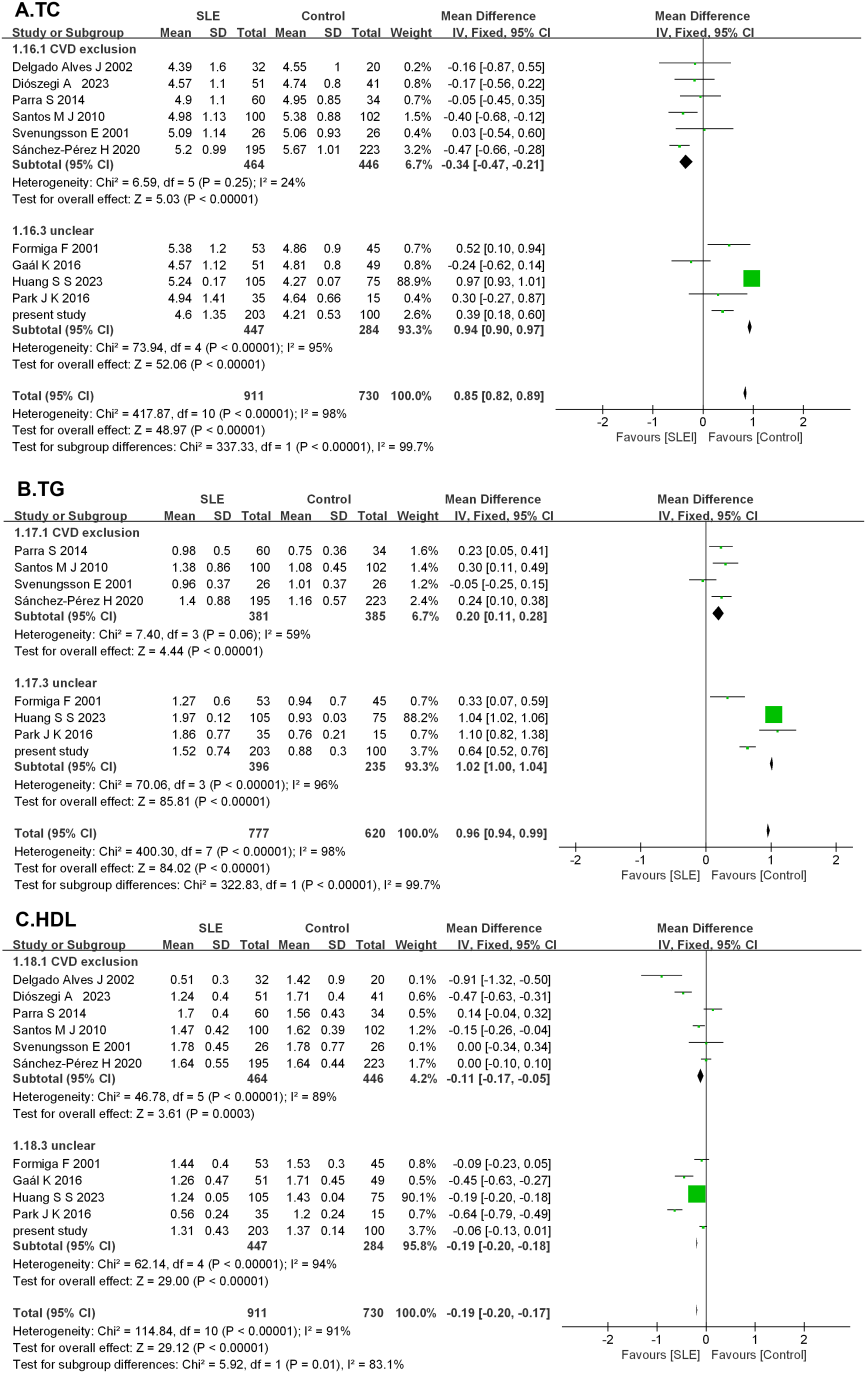
Forest plots in the meta-analysis of serum lipid profile in SLE. **(A)** Forest plots in the meta-analysis of TC in SLE. **(B)** Forest plots in the meta-analysis of TG in SLE. **(C)** Forest plots in the meta-analysis of HDL in SLE.

A total of 1,363 articles were retrieved through searching in PubMed (*n* = 413), Cochrane (*n* = 29), Embase (*n* = 917), and other sources (*n* = 4). After an initial evaluation of titles and abstracts, 1,272 articles were excluded. Full texts of the remaining 91 studies were assessed, and finally, 10 studies were identified according to the inclusion and exclusion criteria. The characteristics of the included studies are presented in **Table 6**. Together with the present case–control study, 11 studies were analyzed in the meta-analysis, which contained 911 patients with SLE and 730 HCs. After 11 studies pooled into the meta-analysis, serum TC levels were elevated in SLE (MD = 0.85, 95% CI 0.82 to 0.89, *p* < 0.00001). As a significant heterogeneity was observed (*I* ^2^ = 98%), the subgroup analysis was performed based on whether CVD was excluded or unclear in the SLE group. It showed that serum TC levels were decreased significantly in SLE without CVD in six studies (MD = −0.34, 95% CI −0.47 to −0.21, *p* < 0.00001), but elevated considerably in SLE with unclear in other 4 studies (MD = 0.94, 95% CI 0.90 to 0.97, *p* < 0.00001). As for serum TG, eight studies were pooled into a meta-analysis. TG was elevated in SLE (MD = 0.96, 95% CI 0.94 to 0.99, *p* < 0.00001). Subgroup analysis also showed elevated TG levels in SLE either without CVD (MD = 0.20, 95% CI 0.11 to 0.28, *p* < 0.00001) or with unclear (MD = 1.02, 95% CI 1.00 to 1.04, *p* < 0.00001). After 11 studies were pooled into the meta-analysis, HDL levels showed reduced HDL levels in SLE (MD = −0.19, 95% CI −0.20 to −0.17, *p* < 0.00001). Subgroup analysis showed lower HDL levels in SLE either without CVD (MD = −0.11, 95% CI −0.17 to −0.05, *p* < 0.00001) or with unclear (MD = −0.19, 95% CI −0.20 to −0.18, *p* < 0.00001). However, significant heterogeneity was still observed (**Figure 4**).

**Table 6:** Eligible study characteristics in the meta-analysis.

Study	Country	Participants (SLE/Control)	Female/Male (SLE, Control)	Serum lipid profile	CVD exclusion	Disease activity (scores)	Disease duration (years)
Formiga F 2001 (25)	Sweden	53/45	Unclear	TC, TG, HDL, and LDL	Unclear	SLEDAI: 4 ± 2	10.5 ± 5.5
Svenungsson E 2001 (26)	Spain	52/26	Unclear	TC, TG, HDL, and LDL	Yes	SLAM: 5.5	19.25 ± 9.7
Delgado Alves J 2002 (27)	Britain	32/20	28/4, 17/3	TC and HDL	Yes	BILAG: 6.7 ± 4.5	5.8(1-12.3)
Santos M J 2010 (28)	Portugal	100/102	Unclear	TC, TG, HDL, and LDL	Yes	SLEDAI-2K: 2 ± 4	6.6 ± 6.8
Parra S 2014 (29)	Spain	60/34	Unclear	TC, TG, HDL, and LDL	Yes	SLEDAI <4	Unclear
Gaál K 2016 (21)	Korea	51/49	44/7, 41/8	TC, HDL, and LDL	Unclear	SLEDAI: 4.0 (2.0–6.0)	6.59 ± 5.26
Park J K 2016 (30)	Germany	35/15	34/1, 13/2	TC, TG, HDL, and LDL	Unclear	SLEDAI-2K: 4.26 ± 4.24	12.1 ± 7.6
Sánchez-Pérez H 2020 (31)	Spain	195/223	185/10, 155/68	TC, TG, HDL, and LDL	Yes	SLEDAI: 2.0 (0.0–5.0)	17 ± 10
Diószegi A 2023 (32)	Hungary	51/41	44/7, 36/5	TC, HDL, and LDL	Yes	SLEDAI: 5.96 (2–10)	Unclear
Huang S S 2023 (33)	China	105/75	94/11, Unclear	TC, TG, HDL, and LDL	Unclear	SLEDAI: 10.70 ± 0.57	6.10 ± 0.62
**Present study**	China	203/100	179/24, 90/10	TC, TG, HDL, and LDL	Unclear	SLEDAI: 5.9 ± 5.3	Unclear

## 4 Discussion

The association between metabolic disorders and autoimmune diseases has received growing attention recently (34), while the association between dyslipidemia and SLE is not yet well defined. To better understand it, the serum lipid profile was first performed in patients with SLE. The results showed that the concentrations of TC and TG increased significantly in patients with SLE compared to HCs, while HDL decreased. LDL did not show a statistical difference in patients with SLE compared to HCs. The subsequent meta-analysis also displayed the same results. Considering the effects of traditional factors (e.g., age and sex) on lipid profile, the adjusted results showed that LDL was also increased significantly in patients with SLE.

Probably, LDL is easily oxidized to oxLDL, which plays a key role in the metabolic disorders in patients with SLE. One study indicated no significant difference in the LDL level of patients with SLE with metabolic syndrome compared to HCs, but the oxLDL level was significantly increased (12). Paraoxonase1 (PON1) activity was evaluated in patients with SLE because it protects LDL from oxidation, its activity was compromised, and the antioxidant capacity of LDL was sequentially impaired. This resulted in an increasing possibility of atherosclerosis. oxLDL accumulation is mainly attributed to HDL dysfunction, reduced PON1 activity, and increased susceptibility of LDL to oxidation (30). Multiple factors such as corticosteroid usage, overweight, and age are responsible for increasing LDL oxidative susceptibility (20). One study reported that HCQ reduced LDL levels and promoted the conversion of TC to HDL (35). However, changes in LDL in patients with SLE need to be further confirmed in the future.

SLE is nine times more common in young women than in men. Patients with SLE have a risk of atherosclerosis almost twice as high as HCs compared by age and sex (36). Patients with SLE had a 27% higher risk of non-fatal CV events compared with age- and sex-matched patients with diabetes (37). Hence, the effect of sex differences on lipids was analyzed in the SLE group and HC group. The results present heterogeneity. Male individuals had higher LDL levels than female individuals in the SLE group. In support, multiple studies showed that being male was associated with higher mortality in SLE (38, 39). A multicenter study found that approximately 25% of 10,000 deaths with SLE were caused by CVDs (40). It was reported that patients with SLE had a 50-fold higher risk of CVDs than matched controls (41). The sex hormone changes may also be associated with increased cardiovascular risk in menopausal women (42). In addition, age-specific metabolomic profiles were identified in patients with SLE vs. HCs, reduced HDL, and elevated GlycA levels associated with disease activity, atherosclerosis, and myocardial infarction at all ages. Glycolysis pathway metabolites uniquely increased with age in SLE, significantly influenced by prednisolone and HCQ treatment (43). Genetic factors are one cofactor of SLE etiopathogenesis and the risk factor of dyslipidemia. Apart from the sex and age of the patients, autosomal mutations of the LDL receptors cause familial hypercholesterolemia (44). Another study reported that CC homozygosity of the GCKR gene and plasma TG concentrations are independently associated with subclinical carotid atherosclerosis in women with SLE (45).

HCQ and GCs are frequently used in the clinic in SLE pharmaceutical therapy. In this study, HDL was increased in patients with SLE with medications and HCQ compared to others without medications. TC was also elevated in patients with SLE with HCQ and or GCs. Moreover, an increase in TG was accompanied by GC use. Multiple linear regression analysis was performed to explore medications’ effects on lipid profile in SLE, combined with other factors (age, sex, and disease duration). The results showed that GC use was an independent risk factor of elevated TC and LDL, and HCQ use was an independent risk factor of increased HDL. They are consistent with other published reports. HCQ is the milestone of SLE therapy for its multiple beneficial effects, including anti-inflammatory and lipid-lowering properties and controlling the glycemic profile. HCQ has the following atheroprotective effects: (i) NO (nitric oxide) availability increases; (ii) improvement in the function of endothelium by reduction of ROS; (iii) reduction of type I IFN production by pDCs; (iv) an impediment to NET formation; and (v) inhibition of platelet activation and aggregation (46). Current evidence and previous studies have provided promising therapy for HCQ on CVD burden in SLE. GCs are anti-inflammatory drugs widely used in many autoimmune inflammatory diseases, including SLE and RA. GCs are essential in relieving pain and reducing the risk of joint injuries. Long-term GC administration increases the risk of dyslipidemia and premature CVDs (47). GCs impact multiple aspects of lipid metabolism, including (i) promotion of insulin resistance, (ii) increase in VLDL from hepatic synthesis, (iii) reduction in LPL, (iv) increase in the conversion of VLDL to LDL, (v) downregulation of the LDL, and (vi) increase in the activity of 3-hydroxy-3-methylglutaryl coenzyme A. These effects on GCs develop hypertriglyceridemia (48).

The present study shows a strong correlation between serum lipids and active SLE. Increased TG and decreased HDL were related to SLEDAI. Decreased HDL was particularly associated with disease progression indicators such as decreased WBC, C3, and C4 consumptions and increased IgG, IgA, CRP, ESR, ANA titers, anti-dsDNA, and anti-C1q. TG significantly correlates with WBCs, lymphocytes, neutrophils, and monocytes. Similar results were seen in our study. However, the hypothesis that serum lipids are the causal pathway for leukocytogenesis is not yet tenable because leukocyte development is influenced by multiple factors (49). Increased TC was associated with kidney damage 24-h PRO, PRO, ACR, and the inflammatory marker CRP. 24-h PRO was also positively correlated to TG and HDL. There is evidence that hyperlipidemia in patients with SLE may be involved in their kidney injury through TNFSF1A and TNFSF1B (33). Hence, abnormality of serum lipids could serve as an indicator reflecting renal injury.

Further analyzing the association between serum lipids and clinical features in the dyslipidemia of patients with SLE proved that dyslipidemia is linked to disease progression. Notably, increased TC was correlated with elevated SLEDAI scores and serious renal damage in patients with dyslipidemia. In contrast, decreased HDL was associated with elevated TNFα, and increased TG was accompanied by elevated CRP. The exact mechanism by which inflammation reduces HDL levels is yet unclear. Nonetheless, TNFα was increased in patients with SLE with CVDs two to three times higher than those without CVDs (50). Patients with SLE have a high prevalence of metabolic syndrome; 56% of patients over 55 years old exhibited hypercholesterolemia, which is directly attributed to the inflammatory status and increased oxidative stress (20, 51). The systemic inflammatory state of lupus disrupted cholesterol homeostasis and increased the susceptibility of arterial wall cells (including macrophages and endothelial cells) to cholesterol accumulation (52). Cholesterol accumulation may exacerbate T- and B-cell responses to facilitate autoantibody production in patients with SLE (53). Reduced HDL may cause cholesterol accumulation through impaired cholesterol efflux capacity (CEC) (54). HDL displays many biological functions, including cholesterol mobilization, antioxidants, anti-inflammatory, anti-thrombotic, and anti-apoptotic effects (55). Meanwhile, lipid-related CV risk in autoimmune diseases has been attributed to inflammation-induced HDL dysfunction. HDL dysfunction leads to anti-inflammatory HDL converting to the phenotype of proinflammatory HDL (56–61). The pro-inflammatory HDL in female patients with SLE has been associated with carotid artery atherosclerosis and increased carotid intima-media thickness (59). Increases in proinflammatory HDL were correlated with increases in oxLDL in SLE. They formed a vicious cycle including inflammation, the formation of proinflammatory HDL, and increased oxLDL (61). In our study, decreased HDL was associated with increasing risks of kidney, lung, digestive, joint, and blood disorders and skin damage. The evidence showed that increased CRP was linked to LPL, the rate-limiting enzyme in TG metabolism. This activity might correlate with autoantibodies in SLE (52). It sequentially reduces the ability to remove chylomicrons from plasma, accumulating chylomicrons and VLDL and ultimately elevating TG level (62). Elevated TG levels may affect vascular function and contribute to vascular inflammation in patients with SLE (32). Inflammation damages and impairs organ function if it is excessive and dysregulated (63, 64). In the cardiovascular system, atherosclerosis is the prominent impact of inflammation (65, 66). Endothelial dysfunction is the beginning of atherosclerosis development in SLE. The mechanism of the endothelial lesion is the disruption of local microenvironmental homeostasis through multifactorial oxidative stress, pro-inflammatory cytokines, NETs, the activation of B cells and autoantibodies, and abnormal T cells. Mitochondrial dysfunction, energy metabolism, and telomere alterations contribute at a molecular level to the increased oxidative stress in SLE. oxLDLs can stimulate endothelial activation. High levels of oxLDL and proinflammatory HDL reduced LDL uptake capacity in SLE and coronary or peripheral arterial disease, and carotid plaque was found (67, 68).

The atherosclerotic plaque was formed via at least three events. Endothelial dysfunction may be primarily present in endothelial lesions, caused by an imbalance between vasoconstrictive (endothelin) and vasodilating (NO) substances and the formation of ROS in the damaged endothelial cells. Inadequate NO synthesis triggers the production of proinflammatory cytokines, including TNFα, IL6, and IL1β, and reduces the production of antithrombotic agents in the endothelium. Thereby, LDL accumulates in the vascular wall. Subsequently, the accumulated LDL in the arterial intima is modified (aggregation, oxidation, and/or glycosylation) and converted into oxLDL; the release of chemokines is necessary for the recruitment of monocytes (CCL2 and CCL5) and T lymphocytes from the bloodstream to the intima. The inflammatory process further accelerates the recruitment and activation of neutrophils, monocytes, and T and B lymphocytes towards the lesion. Finally, the atherosclerotic plaque formed, including rupture, thrombosis, or bleeding. Additionally, platelets, which tend to stick to the intima, contribute to the growth of the atheromatous plaque (69).

One apparent phenotype of SLE is various autoantibody productions such as anti-dsDNA, anti-C1q and anti-Sm, anti-neutrophil cytoplasmic antibodies (ANCA), and antiphospholipid antibodies. Consequently, the immune complexes binding with C1q interrupted cholesterol homeostasis in the arterial wall by inhibiting the expression of two reverse cholesterol transport proteins (CYP27A1 and ABCA1) involved with cholesterol efflux in macrophages for the formation of foam cells (70). Additionally, HDL regulates cholesterol reverse transport. The above explained the phenomenon that decreased HDL was associated with elevated anti-dsDNA and anti-C1q antibodies.

In metabolism studies in patients with SLE, the often-mentioned antibodies to cardiolipin (aCLs) and β2 glycoprotein1 (β2-GP1) are correlated with decreased HDL and ApoA1 (71). A large complex is formed by autoantibodies to β2-GP1 binding to oxLDLs, and the complexes can be taken up by macrophages with the scavenger receptors, by which macrophages are eventually converted to foam cells (72). However, there is no correlation between serum lipids and aCL or anti-β2-GP1 in our study. The possible reason is that total aCL and anti-β2-GP1were measured instead of IgG and IgM aCL and anti-β2-GP1.

The association between serum lipids and SLE cannot directly elucidate the mechanisms causing the metabolic disorder. This should be addressed. Furthermore, apparent heterogeneity was observed in the studies evaluated. Disease duration, CVD exclusion, and the use of lipid-altering drugs showed differences across included studies, which may contribute to this heterogeneity. Meanwhile, there are inherent differences in the study populations, including demographics, gender, ethnicity, and geographic location, and variations in the criteria for inclusion may also contribute to heterogeneity and weaken the strength of the meta-analysis. Based on the above, subgroup analyses were performed to determine whether the included studies combined CVD or not, and the final results were consistent with the results of this study.

In conclusion, this study provided strong evidence for the association between serum lipids and disease activity in patients with SLE. Additionally, dyslipidemia may be a cause of disease aggravation in SLE. Serum lipids in SLE pathogenesis and their implications for clinical diagnosis and treatment require further investigation and confirmative evidence.

## Data Availability

The original contributions presented in the study are included in the article/supplementary material. Further inquiries can be directed to the corresponding authors.
